# Supplementary material for: The L-lactate dehydrogenase LldD contributes to oxidative stress resistance, survival from neutrophils, and host colonization in Neisseria gonorrhoeae
Source: Infect Immun. 2026 Jan 30;94(3):e00644-25. doi: 10.1128/iai.00644-25 (PMC12974116; doi:10.1128/iai.00644-25)
Supplement: Supplemental figures — Fig. S1 to S7. [file iai.00644-25-s0002.docx]

**Title:** The L-lactate dehydrogenase LldD contributes to oxidative stress resistance, survival from neutrophils, and host colonization in *Neisseria gonorrhoeae*

**Authors:** Jerri M Lankford^a^, Willis E Barr^a^, Cole A Andersen^a^, Amitha A Karuppiah^a^, Keena S Thomas^b^, Ian J Glomski^b^, Wen-Chi Huang^a^, Alison K Criss^b^, Aimee D Potter^a^#

^a^Department of Microbiology and Immunology, University of Iowa Carver College of Medicine, Iowa City, Iowa, USA

^b^Department of Microbiology, Immunology, and Cancer Biology, University of Virginia, Charlottesville, Virginia, USA

**Running Head:** LldD mediates ROS resistance and host colonization

#Address correspondence to Aimee D. Potter, aimee-potter@uiowa.edu.

**Supplemental Material:**

Fig S1

Fig S2

Fig S3

Fig S4

Fig S5

Fig S6

Fig S7

Supplemental Dataset 1

 
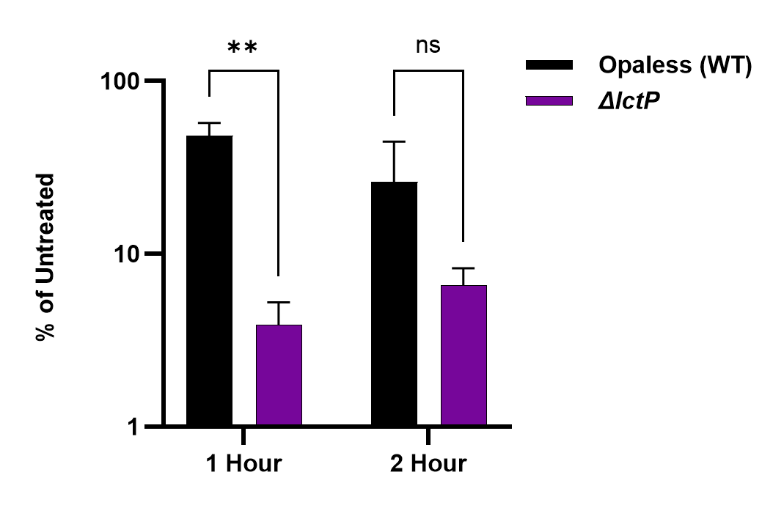


**Fig S1:** **Lactate permease is required for full resistance of Gc to paraquat derived superoxide.** WT and Δ*lctP* Gc were exposed to 50 µM paraquat in GCBL. CFU were enumerated post-exposure at the indicated timepoint. Bacterial burden is reported relative to the corresponding untreated strain at that timepoint (100%). Bars represent the mean. Error bars represent SEM. (A and B) n = 3-5 biological replicates. WT replicates represent a subset of data reported in Fig. 2A and B. Significance determined by mixed effects model with Holm-Šídák’s multiple-comparison test on log-transformed data. **, *P* < 0.01. ns= not significant.

**
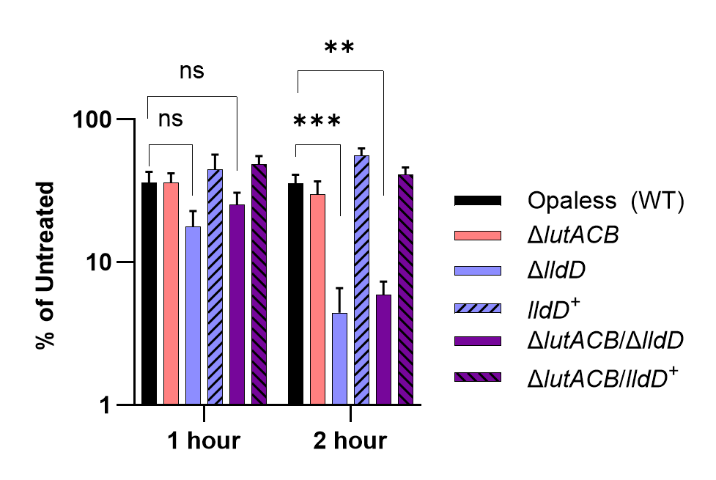
**

**Fig S2:** **The L-lactate dehydrogenase LldD supports Gc resistance to H_2_O_2_.**  WT, Δ*lutACB,* Δ*lldD,* Δ*lldD+,* Δ*lutACB/lldD,* and Δ*lutACB/lldD*+ Gc were exposed to 24 mM H_2_O_2_ in GCBL. CFU were enumerated after 1 h or 2 h. Bacterial survival is reported relative to the corresponding untreated strain at the same timepoint (set to 100%). Bars represent the mean. Error bars represent SEM. n= 3-4 biological replicates. Significance determined by mixed effects model with Holm-Šídák’s multiple-comparison test on log-transformed data .**, *P* < 0.01; ***, *P* < 0.001;. ns= not significant.


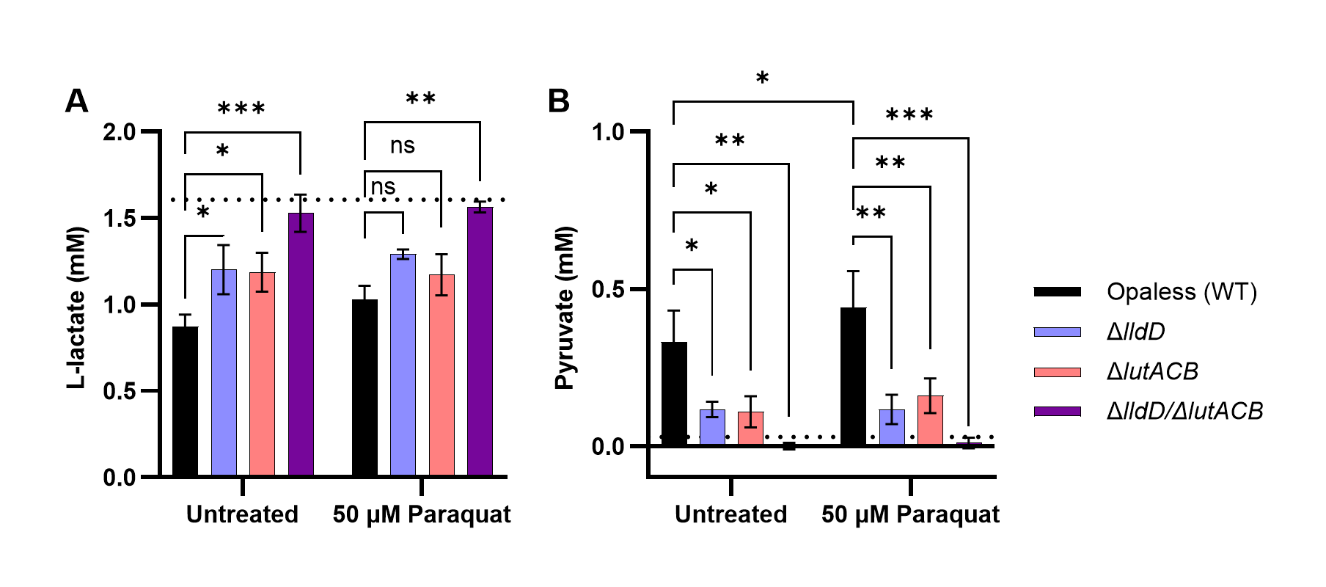


**Fig S3:** **Consumption of L-lactate and production of pyruvate is largely unchanged with exposure to paraquat.** WT Gc, Δ*lutACB,* Δ*lldD,* and Δ*lutACB/lldD* were exposed to 50 µM paraquat in GCBL. Bacteria were collected, pelleted, and supernatants were retained for measurement of (A) L-lactate and (B) pyruvate. Bars represent the mean. Error bars represent SEM. Dotted line indicates baseline metabolite concentration in media at time 0. n = 3 biological replicates. Significance determined by mixed effects model with Holm-Šídák’s multiple-comparison test. ns = not significant. No comparisons between untreated and paraquat treated were significant unless indicated. *, *P* < 0.05; **, *P* < 0.01; ***, *P* < 0.001; ****.


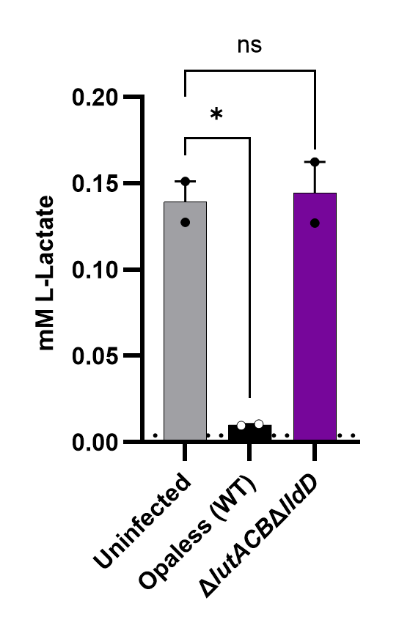


**Fig S4.** **Production of L-lactate by PMNs.** IL-8 stimulated PMNs were left uninfected or exposed to Opaless WT Gc or Δ*lutACB/lldD* for 2 hours in RPMI without FBS. Supernatants were collected, pelleted to remove cells and cell debris, and retained for measurement of L-lactate. Bars represent the mean. Error bars represent SEM. Dotted line indicates baseline metabolite concentration in media at time 0. n = 2 biological replicates from different donors. Significance determined by mixed effects model with Holm-Šídák’s multiple-comparison test. ns = not significant. *, *P* < 0.05.


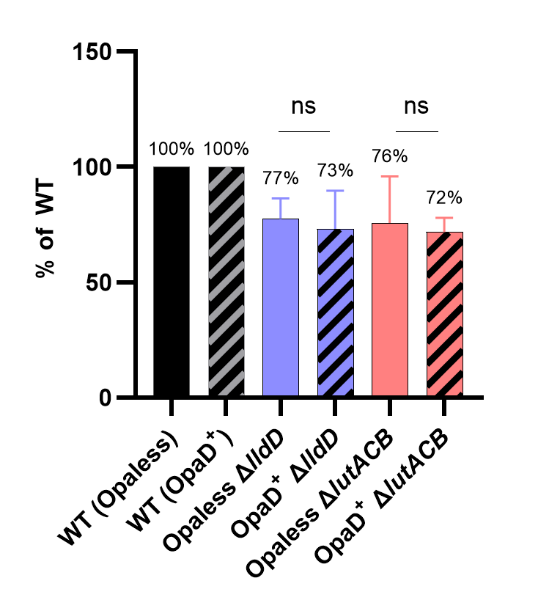


**Fig S5.**  **L-lactate dehydrogenases contribute to bacterial survival from PMNs equally in Opaless and Opa expressing Gc.** WT, Δ*lutACB,* and Δ*lldD* Gc in the Opaless strain background or an isogenic non-variable OpaD^+^ strain were inoculated into IL8-treated PMNs in suspension.. Percent of WT survival at 2 hours was calculated by enumerating CFUs from PMN lysates or media alone and reporting as % CFU relative to the respective strain. n=4-9. Significance was determined by mixed effects model with Holm-Šídák’s multiple-comparison test. No significant difference between Opaless and OpaD^+^ strains was detected.


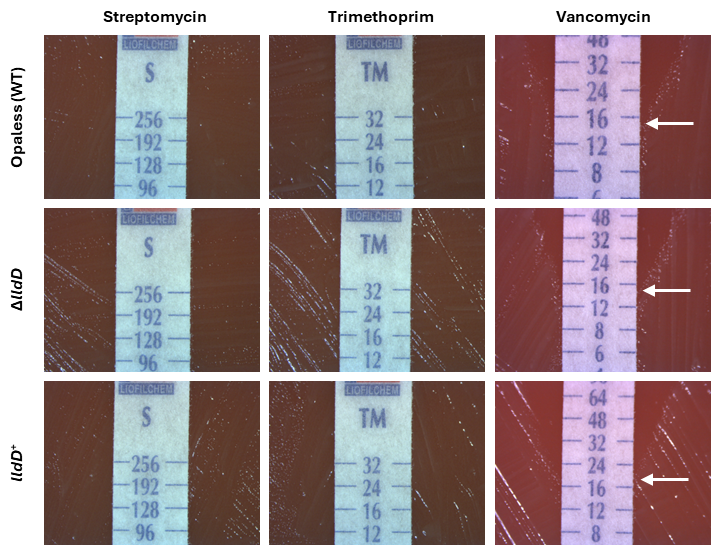


**Fig S6.** Antibiotic susceptibility of Opaless (WT), Δ*lldD,* and *lldD^+^* Gc to streptomycin (0.016-256 μg/mL), trimethoprim (0.002-32 μg/mL), and vancomycin (0.016-256 μg/mL). Minimum inhibitory concentration (MIC) was determined using antibiotic test strips applied to Gc lawns from ~20 isolated colonies incubated for 16 hours on chocolate agar. No zone of clearance was observed for any strain on streptomycin or trimethoprim at the maximum concentration tested. MIC of vancomycin was ~16 μg/mL for all strains.


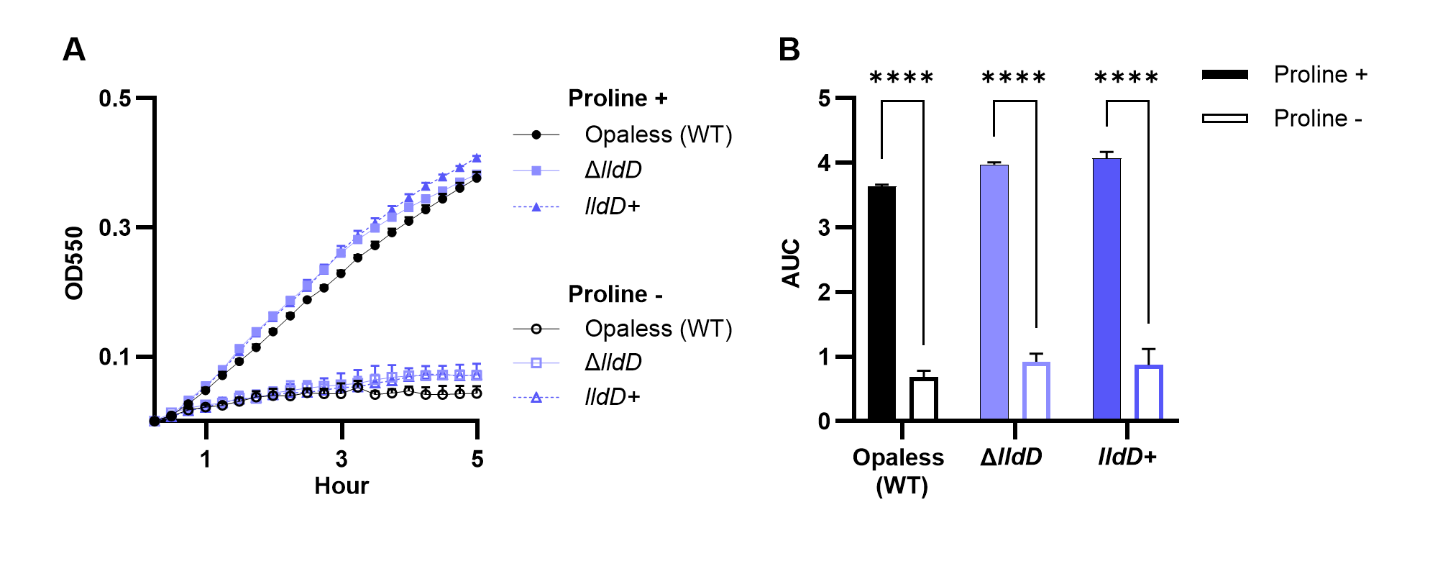


**Fig S7. Growth analysis of Gc in the presence and absence of proline.** WT Gc, isogenic Δ*lldD*, and *lldD+* mutants were cultured in MDM with and without proline. (A) Growth over 5 hours was monitored by optical density at 550 nm for n = 3 biological replicates. (B) Area under the curve (AUC) relative to 0h was calculated for each replicate. Significance determined by repeated measures two-way ANOVA with Holm-Šídák’s multiple-comparison test. Symbols and bars represent the mean. Error bars represent SEM. ****, *P* < 0.0001.
